# Supplementary figures and images for: Selective Genotyping and Phenotyping for Optimization of Genomic Prediction Models for Populations with Different Diversity
Source: Plants (Basel). 2024 Mar 28;13(7):975. doi: 10.3390/plants13070975 (PMC11013471; doi:10.3390/plants13070975)

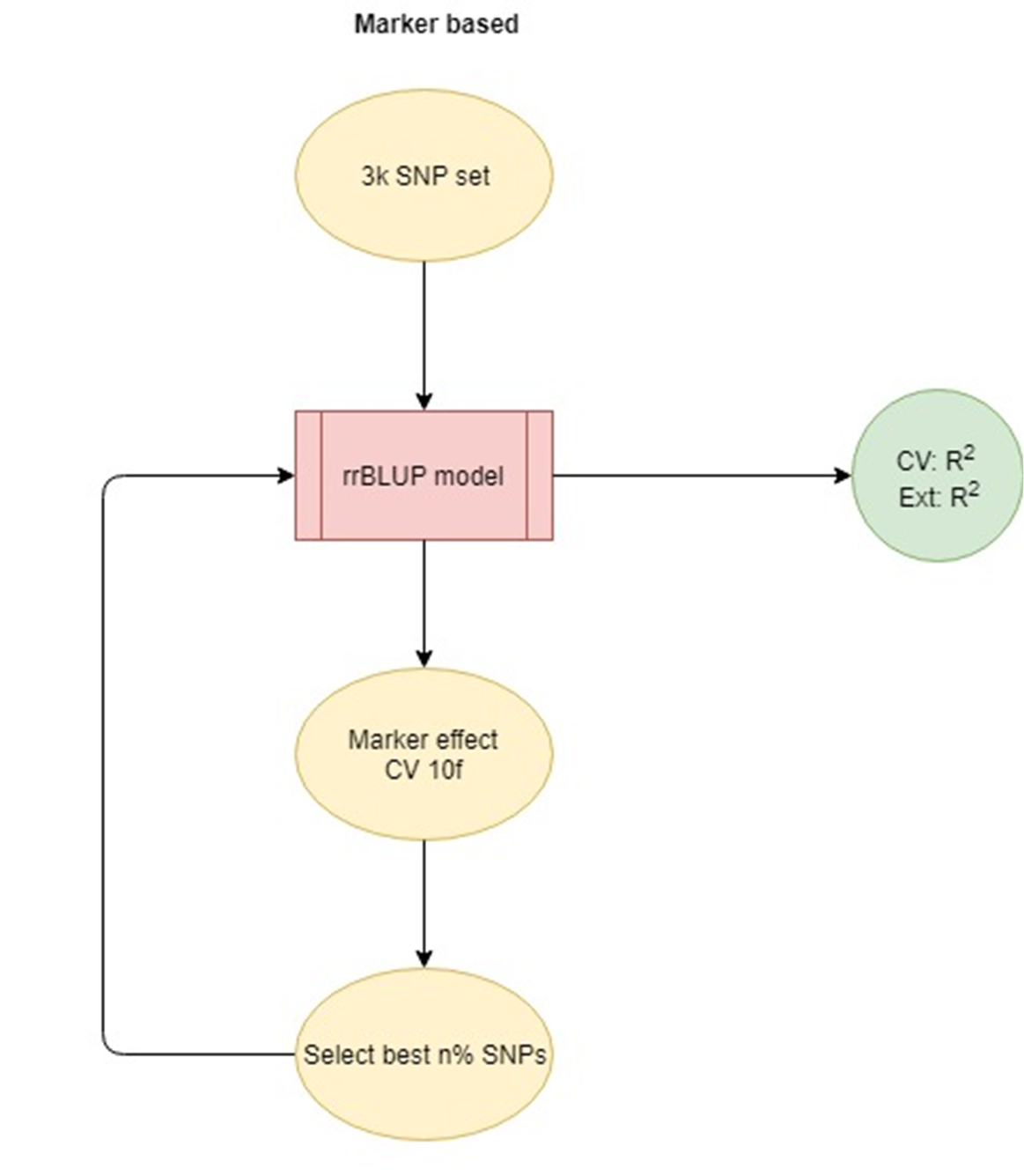

Supplement: Supplementary file 1 [file plants-13-00975-s001.zip › FigureS1a.tif]

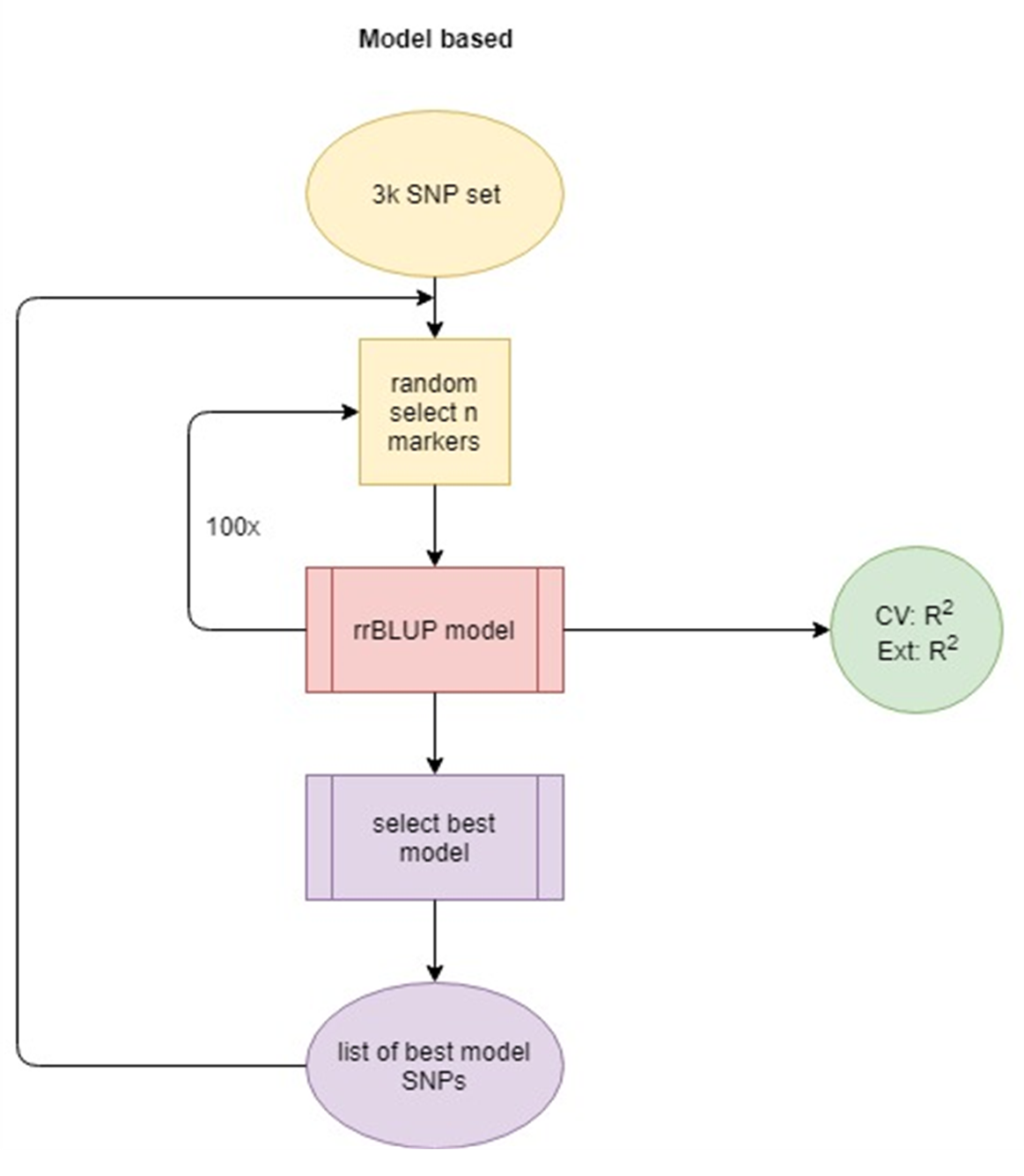

Supplement: Supplementary file 1 [file plants-13-00975-s001.zip › FigureS1b.tif]
